# The arginase inhibitor N^ω^−hydroxy−nor−arginine (nor−NOHA) induces apoptosis in leukemic cells specifically under hypoxic conditions but CRISPR/Cas9 excludes arginase 2 (ARG2) as the functional target

**DOI:** 10.1371/journal.pone.0205254

**Published:** 2018-10-11

**Authors:** King Pan Ng, Aditi Manjeri, Lin Ming Lee, Zhu En Chan, Chin Yee Tan, Qiancheng Darren Tan, A'Qilah Majeed, Kian Leong Lee, Charles Chuah, Toshio Suda, S. Tiong Ong

**Affiliations:** 1 Cancer and Stem Cell Biology Signature Research Program, Duke−NUS Medical School, Singapore, Singapore; 2 Cancer Science Institute of Singapore, National University of Singapore, Singapore, Singapore; 3 Department of Haematology, Singapore General Hospital, Singapore, Singapore; 4 International Research Center for Medical Sciences, Kumamoto University, Japan; 5 Department of Medical Oncology, National Cancer Centre, Singapore, Singapore; 6 Department of Medicine, Duke University Medical Center, Durham, NC, United States of America; University of South Alabama Mitchell Cancer Institute, UNITED STATES

## Abstract

Cancer cells, including in chronic myeloid leukemia (CML), depend on the hypoxic response to persist in hosts and evade therapy. Accordingly, there is significant interest in drugging cancer-specific hypoxic responses. However, a major challenge in leukemia is identifying differential and druggable hypoxic responses between leukemic and normal cells. Previously, we found that arginase 2 (ARG2), an enzyme of the urea cycle, is overexpressed in CML but not normal progenitors. ARG2 is a target of the hypoxia inducible factors (HIF1−α and HIF2−α), and is required for the generation of polyamines which are required for cell growth. We therefore explored if the clinically-tested arginase inhibitor N^ω^−hydroxy−nor−arginine (nor−NOHA) would be effective against leukemic cells under hypoxic conditions. Remarkably, nor−NOHA effectively induced apoptosis in ARG2-expressing cells under hypoxia but not normoxia. Co-treatment with nor−NOHA overcame hypoxia-mediated resistance towards BCR−ABL1 kinase inhibitors. While nor−NOHA itself is promising in targeting the leukemia hypoxic response, we unexpectedly found that its anti-leukemic activity was independent of ARG2 inhibition. Genetic ablation of ARG2 using CRISPR/Cas9 had no effect on the viability of leukemic cells and their sensitivity towards nor−NOHA. This discrepancy was further evidenced by the distinct effects of ARG2 knockouts and nor−NOHA on cellular respiration. In conclusion, we show that nor−NOHA has significant but off-target anti-leukemic activity among ARG2-expressing hypoxic cells. Since nor−NOHA has been employed in clinical trials, and is widely used in studies on endothelial dysfunction, immunosuppression and metabolism, the diverse biological effects of nor−NOHA must be cautiously evaluated before attributing its activity to ARG inhibition.

## Introduction

The bone marrow is a hypoxic organ with an oxygen saturation of ~1–5% [1, 2]. The latter is a critical feature of the bone marrow microenvironment both for normal and leukemic cells [3, 4]. For normal HSCs, exposure to ambient oxygen (≤21%) is detrimental to their function [5] and *ex vivo* HSC expansion is improved by hypoxia [6], while hypoxia enhances homing and drug resistance of leukemic stem cells (LSC) [7, 8]. The importance of the bone marrow microenvironment to LSC persistence and drug resistance has been extensively reviewed [9, 10]. Mechanistically, the cellular hypoxic responses are regulated by two transcriptional factors, the hypoxia inducible factors (HIF-1α and HIF-2α). Compared with normal HSCs, CML and acute myeloid leukemia (AML) LSCs are more dependent on HIF-regulated pathways, and consequently are susceptible to HIF inhibition [11–15]. However, despite the above, and because of the conflicting results from various preclinical studies, direct targeting of HIFs in patients remains controversial [16, 17]. Instead, targeting pathways selectively regulated by hypoxia in leukemic but not normal cells would be more desirable.

Mammalian cells express two arginases, ARG1 and ARG2, which both convert arginine to urea and ornithine, completing the last step of the urea cycle [18]. Ornithine is the precursor of polyamines which is required for cell growth and differentiation. Despite their functional similarity, ARG1 and ARG2 have different cellular localization, and are phenotypically distinct [19–21]. ARG1 is specifically expressed in liver and myeloid cells, while ARG2 is expressed in various cell types including renal cells, endothelial cells, neurons and macrophages [19]. Through depletion of extracellular arginine, arginases are known for mediating immunosuppression in various processes including inflammation, infection and carcinogenesis [22–27]. Additionally, arginases have been found to be overexpressed and drive proliferation in several cancers [27–30]. Because arginine metabolism is central to the above processes, various inhibitors had been developed to modulate arginase activity [31]. Among them, N^ω^-hydroxy-nor-arginine (nor−NOHA) has been widely used as a specific arginase inhibitor, including in clinical trials [32–34], although its specificity has not been vigorously evaluated.

We have recently shown that hypoxia confers resistance towards tyrosine kinase inhibitors (TKIs) and induces the specific expression of ARG2 in CML compared to normal progenitors [15]. Given its potential role in driving tumorigenesis, and the significantly greater induction in CML cells (at least 5-fold higher) [15], ARG2 might therefore represent a druggable target and therapeutic window in hypoxic CML progenitors compared to normal progenitors. We therefore explored the feasibility of targeting arginase in leukemia. We found that the ability to induce ARG2 under hypoxia predicted sensitivity to nor−NOHA in CML cells as well as a broad range of other hematologic malignancies. Surprisingly, ablation of ARG2 using CRISPR/Cas9 did not affect proliferation or sensitivity towards nor−NOHA, implying that the anti-leukemic effect of nor−NOHA is off-target. Our results demonstrate two important findings: the potential for pharmacologic targeting of the differential hypoxic response of leukemic versus normal progenitors, and the need for robust confirmation of putative targets regardless of their biological plausibility.

## Material and method

### Clinical samples

Primary CP and BC CML samples were obtained from patients at the Singapore General Hospital. Written informed consent was obtained from all patients under protocols approved by the Singhealth Centralized Institutional Review Board (CIRB) for use in the current study, in accordance with the Declaration of Helsinki. Cord blood samples were obtained from the Singapore Cord Blood Bank. Mononuclear cells were isolated from patient whole blood using Ficoll−Paque (Sigma-Aldrich) density centrifugation. CD34^+^ cells were purified using a CD34 microbead kit (Miltenyi Biotec, Germany) and magnetic column-based separation.

### Cell lines and reagents

Leukemia cell lines were obtained from the American Type Culture Collection (HL60, KG1, CCRF, KOPTK, U266 and RPMI 8226) and the German Collection of Microorganisms and Cell Cultures (KCL22, K562) and were tested free of mycoplasma by RT−PCR using primers specific to mycoplasma genes. Primary cells were cultured in StemPro−34 SFM (Gibco, Thermo Fisher Scientific) supplemented with 2mM ʟ−Glutamine (Gibco, Thermo Fisher Scientific) and cytokines (GM−CSF 200pg/mL; G−CSF 1000pg/mL; SCF 200pg/mL; LIF 50pg/mL; MIP−1α 200pg/mL; and IL−6 1000pg/mL) [15, 35]. Cell lines were cultured in RPMI-1640 media (Nacalai Tesque) supplemented with 10% FBS (Biowest), penicillin/streptomycin and 2mM ʟ-glutamine (Gibco, Thermo Fisher Scientific). Hypoxic treatment (0.5–2% oxygen, 5% carbon dioxide) was performed in a humidified incubator (I-Glove, Biospherix) or with the use of the hypoxia mimetic CoCl_2_ at 150 μM. Stock solutions of Imatinib (Novartis, Switzerland) were made by dissolving in DMSO/PBS (1/1, v/v) at 1mM. Nor−NOHA (Cayman chemicals, USA) was dissolved in DMSO at 0.25M. Ornithine and arginine (Sigma-Aldrich) was dissolved in H_2_0 at 0.5M.

### Arginase activity assay

Arginase activity was analysed as described [36] with modifications. Cells were counted, and equal numbers of cells were lysed in 50μl of lysis buffer (PBS with 1mM EDTA, 0.1% Triton X−100 and protease Inhibitors) and centrifuged for 15 minutes at 14,000g at 4°C. The supernatants were mixed with 50μl of freshly prepared activation buffer (10mM MnCl_2_, 50mM Tris-HCl pH7.5) and 50μl of 0.5M arginine, and heated for 10 minutes at 56°C. Thereafter, 800μl of acidic solution (H_2_SO_4_ (96%)/H_3_PO_4_ (85%)/H_2_O, 1/3/7, v/v/v; all from Sigma–Aldrich) and 25μl of 9% α–isonitrosopropiophenone (in ethanol; Sigma–Aldrich) were added to the mixture and heated for 15 minutes at 100°C. The mixture was allowed to develop colour in the dark. Finally, 250μl was transferred to a 96-well plate for OD measurements at 550nm.

### Western blotting, RT-qPCR, Annexin V Staining and Colony formation assays

Experiments were performed as before [15]. Specifications of the antibodies used for western blotting and primers used for RT-qPCR can be found in the S1 Table and S2 Table respectively.

### Amino acid content determination

Amino acid profiling was done as described [37] in the Duke-NUS metabolomics facility. Equal numbers of cells were suspended in 50% acetonitrile/0.3% formic acid, and 100μL of cell suspension was extracted using methanol. Extracts were obtained with 3M hydrochloric acid in butanol (Sigma–Aldrich), which were then dried and reconstituted in methanol for analysis in a liquid chromatography–mass spectrometer.

### siRNA transfection

About 1x10^6^ cells were washed and resuspended in 100μl Amaxa nucleofection buffer (Lonza). 100nM of the following siRNAs were then added to the mixtures: siARG2 (Ambion, #s1571), siARG1 (Ambion, #s1568), siHIF1−α (Ambion, #s6539), siHIF2−α (Ambion, #s4700), siARNT (Ambion, #s1613), and control siRNA (Ambion, silencer select control siRNA #1). The mixtures were electroporated using the preset K562 ATCC program on the Amaxa nucleofection device (Lonza). Cells were resuspended in 5ml of RPMI media for recovery before further treatments.

### shRNA transduction

We used a modified pLVX-shRNA2 vector (Clontech) for shRNA experiments. The original CMV IE promoter of pLVX-shRNA2 was replaced with a mouse PGK promoter (PCR and cloned by primers 5’–ACACGAATTCTACCGGGTAGGGGAGGCGCT and 5’–ACACCCCGGGCGAAAGGCCCGGAGATGAGG) to generate the pLVX2-shRNA2-PGK vector which drives persistent ZsGreen (enhanced GFP) expression. Oligos carrying short hairpin sequences were cloned into BamHI/EcoRI sites of the pLVX2-shRNA2-PGK vector, and were co-transfected with the packaging vectors pMD2.G, pRSV–REV and pMDLg/pRRE (kind gifts from A/Prof. Takaomi Sanda) into 293T cells using Fugene 6 (Promega). The virus containing supernatant was collected and filtered 48 hours post−transfection, and was used to transduce the target cells by spinoculation (2500 rpm for 1.5 hours), in the presence of 5–8 ug/ml polybrene. The cells were allowed to recover for 3 days, before GFP-positive transduced cells were sorted by FACS. The hairpin sequences can be found in S3 Table.

### CRISPR/Cas9 targeted knockout

The one vector lentiCRISPRv2 system [38] was used to generate the ARG2 knockout clones. Briefly, the vector was digested with BsmBI and cloned with the desired oligos. The lentivirus production followed that of the shRNA transduction experiments described above. Single cell clones were generated by serially diluting the transduced cells to 2.5 cells/ml, and dispensing them into round−bottomed 96-well plates at 200μl/well, in the presence of 1.5μg/ml puromycin. The expanded clones were recovered for characterisation. The sequences of the oligos are detailed in S4 Table.

### Luciferase reporter construction and assays

The ARG2 promoter was cloned into the pGL3–Luc vector and together with the control vector (pGL3–basic–Renilla), co–transfected using Fugene 6 (Promega) into HeLa cells. About 30,000 HeLa cells were plated in 500μl of DMEM in 24-well plates, and the transfected cells were incubated for 48 hours before being assayed using the dual-luciferase assay system (Promega).

### Measurement of cellular respiration by Seahorse Analyzer

0.1x10^6^ K562 cells were plated per well in poly–L–lysine (Sigma–Aldrich)-coated XF–24 well cell culture microplates in XF Assay media supplemented with 4.5 g/L glucose (Sigma-Aldrich) and 1mM sodium pyruvate (Gibco). The cells were spin–immobilized to the microplates at 200g for 1 minute. The cellular oxygen consumption rate (OCR), extracellular acidification rate (ECAR), and photon production rate (PPR) were obtained using an XF24 Analyzer from Seahorse Bioscience. The measurements were performed according to the manufacturer’s instructions, using Oligomycin (Oligo), Carbonyl cyanide–4–(trifluoromethoxy) phenylhydrazone (FCCP) and Rotenone & antimycin A (R/A; all from Sigma−Aldrich) at the specified concentrations. Data was analysed using the Seahorse XF software (Agilent).

### Generation of mutant ARG2 and transfections

The human arginase–dead mutant ARG2–H160F was constructed as followings. A pair of degenerate primers (Forward 5’–ACTTCATCAGGAAATCTCTTT–GGACAGCCAGTTTCAT; Reverse 5’–ATGAAACTGGCTGTCCAAAGAGATTTCCT–GATGAAGT) was used to generate the ARG2–H160F mutant from the ARG2 expression vector pCMV3-ARG2-C-GFPSpark (Sino Biological Inc.) by site-directed mutagenesis. The amplicon was further amplified (Forward 5’–TGGTACCATGTCCCTAAGGG–GCAGCCTCTC; Reverse 5’–GCTGGAGCCAGTGTAGGGTCAAATG), digested with Bsu36I and EcoRV and cloned into the Bsu36I/EcoRV digested pCMV3-ARG2-C-GFPSpark vector. The vectors were transfected into K562 cells using the Amaxa Cell Line Nucleofector Kit V (Lonza). 3μg of plasmid DNA, 1x10^6^ cells and Nucleofector Solution V were mixed in a cuvette and the cuvette was inserted into the Amaxa device, where transfections were performed using Nucleofector Program T-016. The transfected cells were recovered in RPMI for 24 hours before experiments.

### Statistical analysis

Data were reported as mean ± standard error of the mean. Statistical significance was determined by two−tailed Student’s t-test using either Excel or GraphPad Prism 7.

## Results

### CML cells express and upregulate ARG2 under hypoxia

**Fig 1:**
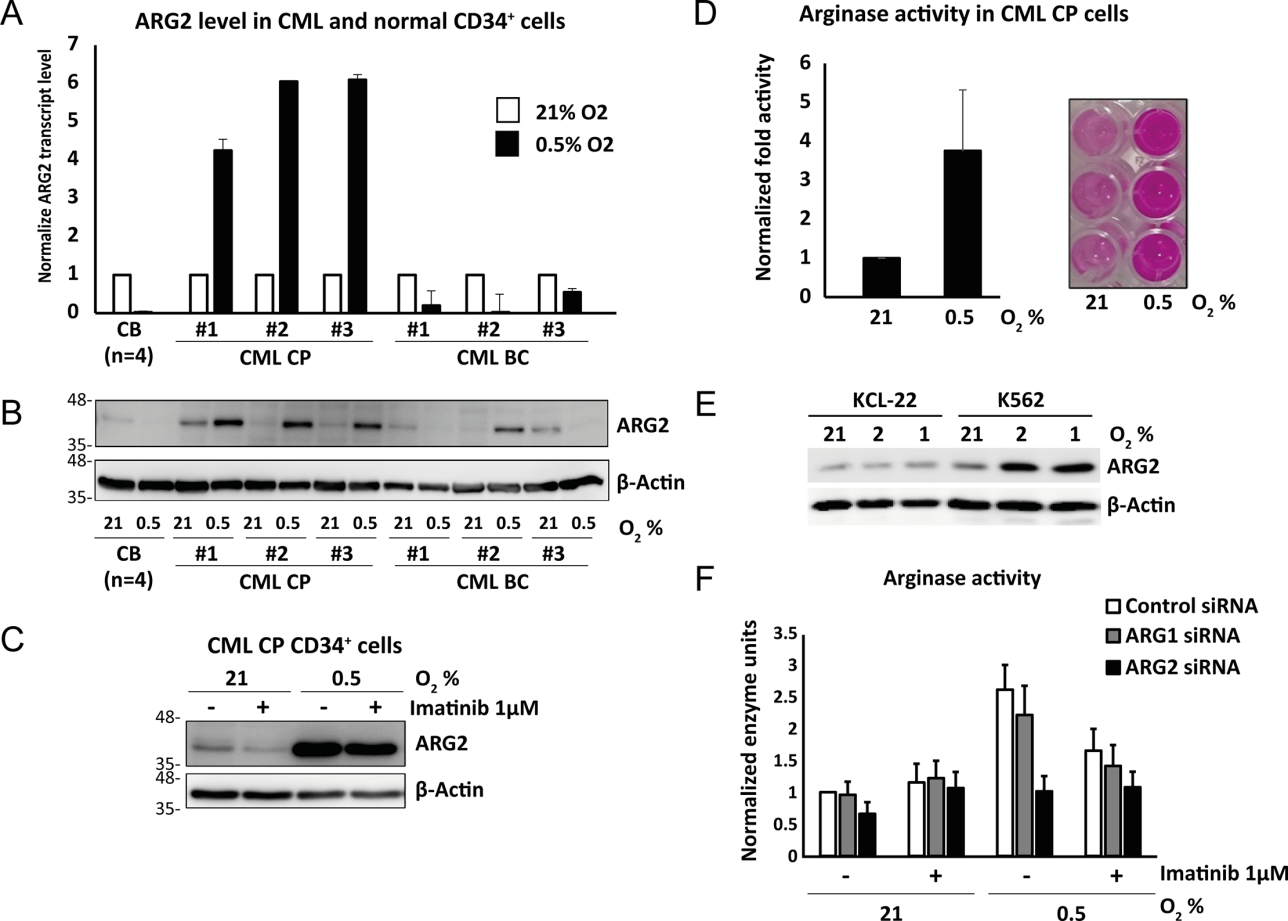
ARG2 is specifically induced by hypoxia in chronic phase CML progenitors. (A) Expression of *ARG2* in cord blood (CB, n = 4, pooled), chronic phase CML (CP, n = 3) and blast crisis (BC, n = 3) CD34^+^ progenitors under normoxia (21% O_2_) or hypoxia (0.5% O_2_) for 96 hours. ARG2 transcript and protein levels were determined by RT-PCR (A) and western blot (B) respectively. (C) ARG2 upregulation persists upon BCR-ABL inhibition. CP CML CD34^+^ progenitors were treated with imatinib (1μM) at 21% or 0.5% O_2_ for 96 hours, then harvested and cell lysates probed for ARG2 protein by western blot. (D) Arginase activity is increased by hypoxia. CP CML CD34^+^ progenitors (n = 2) were incubated under 21% or 0.5% O_2_ for 96 hours, lysed and arginase activity quantified using a colorimetric assay to detect urea production. Right panel shows the representative image of the colorimetric assay. (E) Hypoxia induces expression of ARG2 in CML cell lines. K562 and KCL22 cells were grown at 21%, 2% or 1% O_2_ for 48 hours and the level of ARG2 protein determined by western blot. (F) ARG2 is the dominant arginase in CML. K562 cells were transfected with control, ARG1 or ARG2 siRNA and incubated under 21% or 0.5% O_2_ for 48 hours, before being subjected to arginase activity quantification (average of 4 experiments).

We previously identified a set of genes which were preferentially induced under hypoxia in CML compared to normal CD34^+^ cells, and found that ARG2 induction was at least 5-fold greater in CML cells relative to normal CD34^+^ cells [15]. Arginine is utilized either by arginases or nitric oxide synthases (NOS) to generate ornithine or nitric oxide respectively (Part A in S1 Fig). Since only ARG2 among the arginine metabolizing enzymes (ARGs and NOSs; Part B in S1 Fig) is expressed significantly in CML cells, we focused on the role of ARG2 in CML cells. To confirm the specific expression of ARG2 in CML progenitors, we treated CD34^+^ cells from normal cord blood (CB), chronic phase (CP) and blast crisis (BC) CML samples under normoxia (21% O_2_) or hypoxia (0.5 to 2% O_2_). The oxygen concentration was varied (0.5 to 2% O_2_), but we confirmed the treated cells consistently upregulated ARG2 and proliferated at similar rate across experiments. In line with previous results, hypoxia upregulated ARG2 transcript and protein levels in CML CP cells but not in normal CB cells (Fig 1A and 1B). In primary BC cells and BC CML cell lines, ARG2 expression was more heterogeneous (Fig 1A, 1B and 1E), possibly due to the genetic diversity of BC disease [39]. We also found that imatinib (IM) had some activity in preventing hypoxia-induced ARG2 upregulation (Fig 1C), and that the enzymatic activity of ARG2 correlated with ARG2 protein levels (Fig 1D). Moreover siRNA-mediated knockdown of ARG2, but not ARG1, reduced arginase activity significantly in the CML line K562 (Fig 1F; Part B in S2 Fig). Taken together, we conclude that ARG2 is the predominant arginine metabolizing enzyme in CML cells and it is regulated primarily by hypoxia.

### ARG2 is variably expressed in leukemic cells and is regulated by HIF1-α and HIF2-α

**Fig 2:**
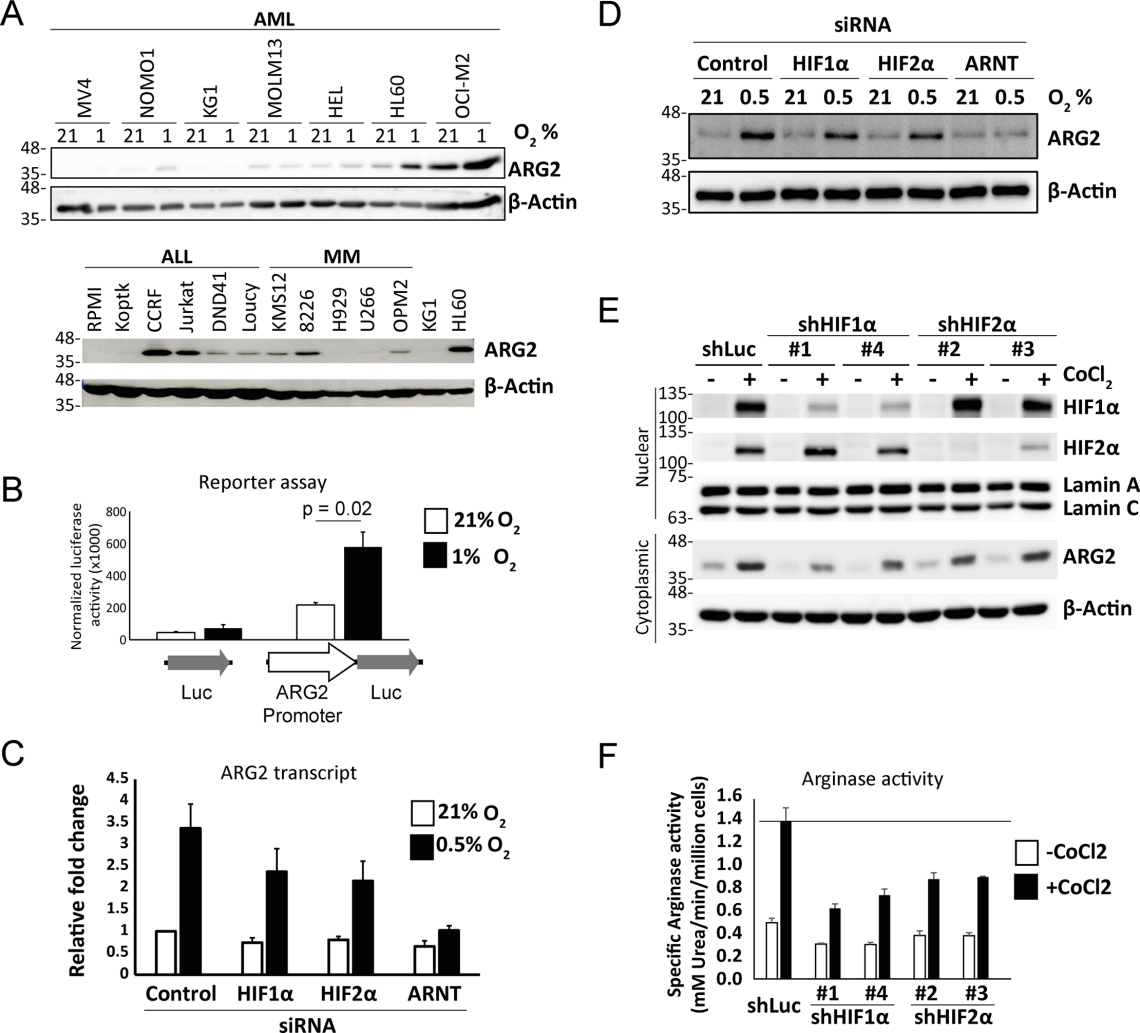
ARG2 is expressed in subsets of hematologic malignancies and is regulated by HIF1-α and HIF2-α. (A) Expression of ARG2 was determined by western blot in a panel of acute myeloid leukemia (AML), acute lymphoid leukemia (ALL) and multiple myeloma (MM) cell lines under 21% or 0.5% O_2_. (B) HIFs regulate ARG2 transcription. Luciferase reporters containing the *ARG2* promoter were transfected into HL60 cells, and incubated under 21% or 1% O_2_ for 48 hours before assaying for luciferase activity (average of 3 experiments). (C) Knockdown of HIF1−α, HIF2−α, or ARNT (HIF1−β) attenuates hypoxic induction of *ARG2*. K562 cells were first transfected with control, HIF1−α, HIF2−α or ARNT siRNA, incubated at 21% or 0.5% O_2_ for 48 hours, following which ARG2 transcript and protein levels were measured by RT−PCR (average of 3 experiments) and western blot (D) respectively. Knockdown of HIF1−α or HIF2−α attenuates hypoxic induction of ARG2 in HL60 cells. HL60 cells were transduced with shRNA-expressing vectors targeting Luc (control), HIF1−α, or HIF2−α. The transduced cells were treated with 150 μM CoCl_2_ for 48 hours and were lysed for (E) western blotting or for (F) arginase activity quantification (average of 4 experiments).

Overexpression of ARG2 has been reported in solid tumours [27–30]. Likewise, ARG2 is expressed at high levels in solid as well as liquid malignancies (Cancer Cell Line Encyclopedia; Part C in S1 Fig). Here, we confirmed the expression of ARG2 in a panel of hematologic malignancies [AML, acute lymphoblastic leukemia (ALL) cell lines and multiple myeloma (MM)]. We found that ARG2 induction by hypoxia, as in BC cells, was heterogenous (Fig 2A). Because HIFs have been reported to regulate arginase expression in various tissues [40–42], we asked if HIFs contribute to the hypoxic induction of ARG2 in CML. The ARG2 gene locus contains several H3K27Ac-enriched regions (enhancer mark), among which only the promoter region contains putative HIF binding sites (predicted by PROMO [43]; Part A in S2 Fig). When cloned into a luciferase reporter, we found the promoter region to be responsive to hypoxia (Fig 2B), suggesting HIFs regulate ARG2 expression. To further confirm the functional roles of HIFs, we knocked down HIF1–α, HIF2–α and ARNT (HIF1–β) in K562 cells using siRNA. ARNT is the constitutively expressed dimerizing partner of both HIF1–α and HIF2–α and it is required for their transcriptional activity. Knockdown of either HIF1−α or HIF2−α partially decreased ARG2 expression, while knockdown of ARNT almost completely abolished ARG2 induction under hypoxia (Fig 2C and 2D and Part B in S2 Fig), suggesting that ARG2 is a downstream transcriptional target of both HIF1−α and HIF2−α. In the AML cell line HL60, knockdown of HIF1–α or HIF2–α by shRNA decreased the expression and activity of ARG2 (Fig 2E and 2F), suggesting HIFs contributed to the expression of ARG2, at least for leukemic cells up−regulating ARG2 under hypoxia. Together, we conclude that both HIF1–α and HIF2–α contribute to the hypoxic induction of ARG2 in leukemia cells.

### The arginase inhibitor nor−NOHA induces apoptosis in ARG2 expressing cells

**Fig 3:**
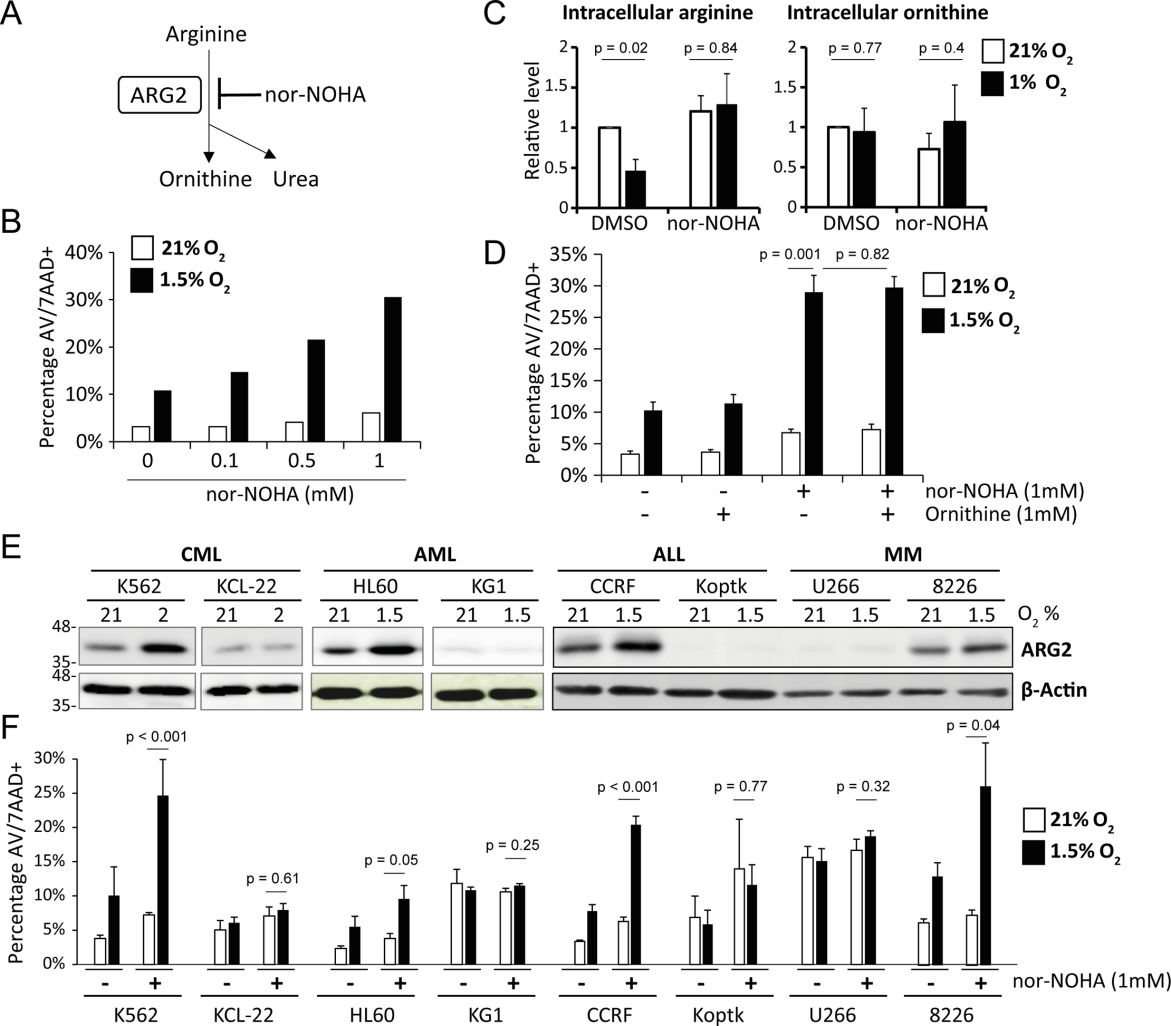
Anti-leukemic activity of the arginase inhibitor nor−NOHA in ARG2-expressing cells. (A) Schematic of cellular arginine metabolism. ARG2 converts arginine into ornithine and urea, a reaction which can be inhibited by the arginase inhibitor nor−NOHA. (B) Anti-leukemic activity of nor−NOHA. K562 cells were treated with vehicle (DMSO) or increasing concentrations of nor–NOHA (0.1, 0.5 or 1mM) under 21% or 1.5% O_2_ for 72 hours, and cell viability determined by Annexin V/ 7−AAD staining. (C) Effect of arginase inhibition on cellular amino acid composition. K562 cells were treated with 500 μM nor–NOHA under 21% or 1% O_2_ for 48 hours. The metabolites were extracted and subjected to quantification by LC−MS (average of 3 experiments). (D) Ornithine does not rescue cell death induced by nor–NOHA. Experiments were as in (B), and ornithine (1mM) was added at the beginning of incubation (average of 3 experiments). (E) Cell lines expressing and upregulating ARG2 under hypoxia are more sensitive to nor−NOHA-induced cell death than those without ARG2 upregulation. Leukemia and myeloma cell lines were grown under 21% or 1.5% O_2_ for 48 hours, and ARG2 expression determined by western blot on cell lysates. (F) The indicated cell lines were grown at 21% or 1.5% O_2_ for 72 hours, and the effect of nor−NOHA on cell viability was analysed by Annexin V/ 7−AAD staining (average of 4 experiments).

The arginase activity of ARG2 can be inhibited by N^ω^–hydroxy–nor–arginine (nor–NOHA; Fig 3A), the most widely used reversible inhibitor of arginase [44]. To probe if the arginase activity of ARG2 contributes to cell survival, we treated ARG2-expressing K562 cells with nor–NOHA, using concentrations at which it is generally used for inhibiting arginase (0.1 to 1mM) [23, 26, 45–47]. While nor–NOHA had little effect on cell viability under normoxia, it significantly induced apoptosis in a dose-dependent manner under hypoxia (Fig 3B). As expected, the level of intracellular arginine was reduced upon induction of ARG2 under hypoxia but was restored by nor−NOHA, confirming the inhibition of arginase activity (Fig 3C). We did not however, observe a significant change in intracellular ornithine levels, which could be due to compensatory pathways for the synthesis of ornithine [18]. As such, addition of ornithine did not rescue nor−NOHA-induced apoptosis (Fig 3D). To determine if hypoxia induction of ARG2 could reliably predict sensitivity towards nor−NOHA treatment, we treated a panel of cell lines that included both high (ARG2-high) and low ARG2 (ARG2-low) expression following hypoxia (Fig 3E). As with K562 cells, ARG2-high cells (HL60, CCRF and RPMI 8226) exhibited increased sensitivity to nor−NOHA under hypoxic compared to normoxic conditions, while ARG2−low cells (KCL22, KG1, KOPTK and U266) did not (Fig 3F). Taken together, we find that ARG2 expression in hematologic malignancies predicts sensitivity to nor−NOHA treatment under hypoxia.

### Nor−NOHA attenuates hypoxia-mediated resistance towards imatinib

**Fig 4:**
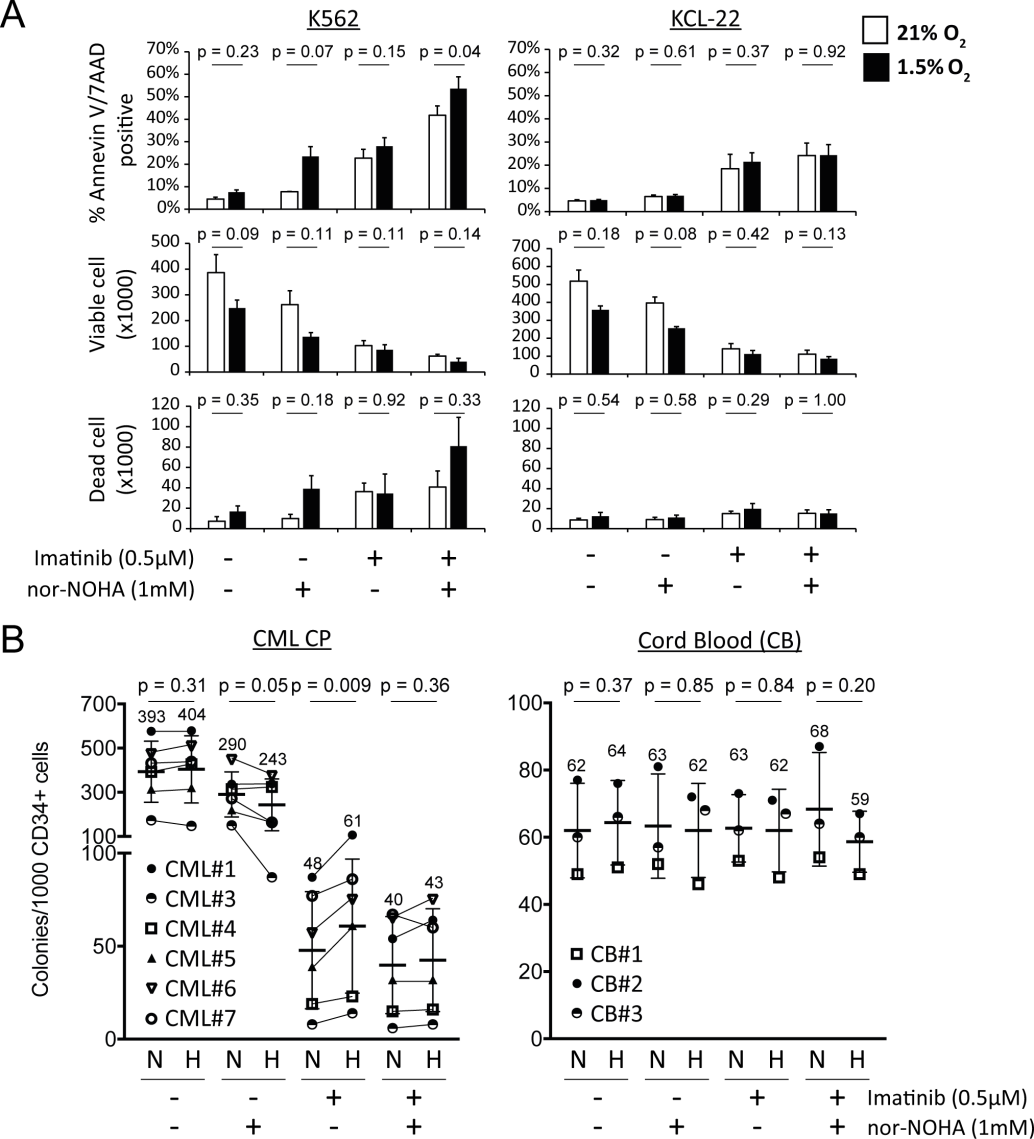
Treatment with nor−NOHA overcomes hypoxia-mediated TKI resistance in CML cells. (A) K562 or KCL22 cells were treated with imatinib (0.5μM) and/or nor−NOHA (1mM) under 21% or 1.5% O_2_ for 72 hours. Cell viability and cell numbers were determined by Annexin V/ 7−AAD staining and flow cytometry (average of 3 experiments). (B) Primary CP CML (n = 6) or normal cord blood (CB; n = 3) CD34^+^ cells were treated with imatinib (0.5μM) and/or nor−NOHA (1mM) at 21% (normoxia; N) or 1.5% O_2_ (hypoxia; H) for 96 hours. The cells were then harvested and plated for colony formation assays (CFA).

We have previously documented that hypoxia confers TKI resistance to primary CML cells [15]. Since nor−NOHA is effective in targeting ARG2-expressing leukemia cells, we wondered if nor−NOHA could attenuate hypoxia−mediated imatinib resistance in CML cells. Although K562 cells differed from primary CP CML cells and did not show resistance towards imatinib under hypoxia (unpublished data), the apoptotic effect of imatinib, marked by the dramatic increase in the number of dead cells, was further enhanced by nor−NOHA co−treatment (Fig 4A). KCL-22 cells, which are known to be resistant to cell death caused by imatinib [48], show no significant change in dead cell numbers whether in single or combined treatments (Figs 3F and 4A). Next, we determined if primary CML cells were also sensitive to combined nor−NOHA and imatinib treatment. CD34^+^ CP−CML cells were treated with imatinib and nor−NOHA, and the effect on colony-forming cells (CFCs) assayed (Fig 4B and S3 Fig). As previously reported [15], more CML CFCs survived under hypoxia upon imatinib treatment. Consistently, nor−NOHA was more effective under hypoxia in 4 out of 6 patients when examined individually (S3 Fig), while, in combination, nor−NOHA successfully overcame the imatinib resistance conferred by hypoxia in 5 out of 6 patients (Fig 4B and S3 Fig). Interestingly, in the same 5 out of 6 primary patient samples, nor-NOHA displayed a therapeutic effect over and above that of imatinib under both normoxia and hypoxic conditions. Both imatinib and nor−NOHA had no significant effect on 3 independent lots of normal cord blood CFCs (Fig 4B), which express little or no ARG2 (Fig 1) indicating the presence of a therapeutic window where both drugs target disease cells specifically. Thus, nor−NOHA is able to overcome hypoxia-mediated TKI resistance in CML progenitors while having little activity against normal progenitors.

### Cell death induced by nor−NOHA is independent of ARG2

**Fig 5:**
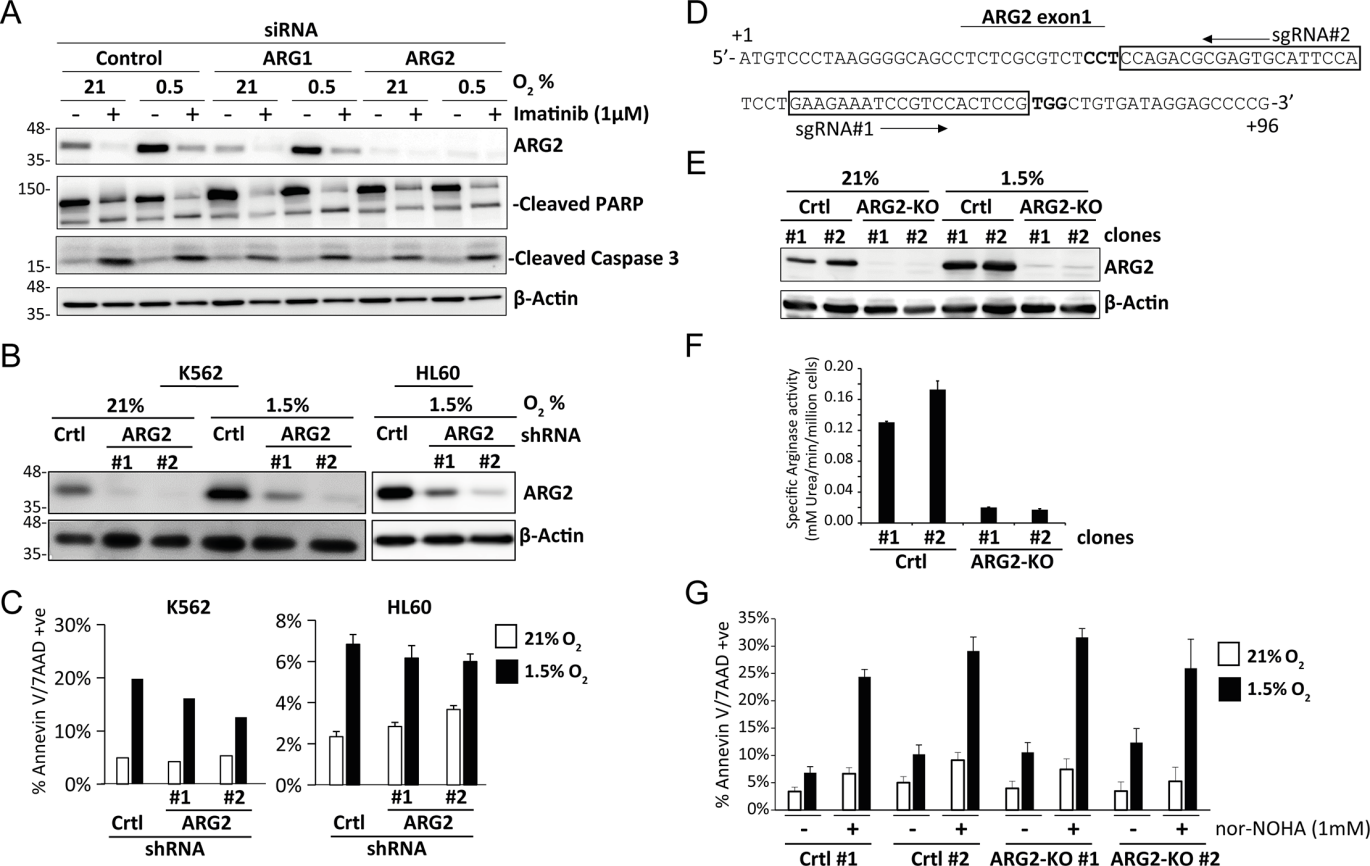
Depletion of ARG2 does not induce cell death or compromise the effects of nor−NOHA. (A) K562 cells were transfected with control, ARG1 or ARG2 siRNA and incubated at 21% or 1% O_2_ for 48 hours, with or without imatinib (1μM). Levels of ARG2, cleaved PARP and cleaved caspase 3 were detected by western blot on cell lysates. K562 and HL60 cells were transduced with shRNA expressing vector targeting Luc (control) or ARG2, and transduced cells were grown at 21% or 1.5% O_2_ for 48 hours (B; western) or 72 hours (C; Annexin V/ 7−AAD staining V) before harvesting for the respective assays. (D) Schematic showing the genomic sequence for a portion of the first exon of ARG2. The targeting sequence of two independent sgRNAs are boxed, with the PAM sequence in bold. The translational initiation site (+1) is marked. (E) Characterization of the ARG2-KO clones generated by CRISPR/Cas9 nuclease. The control (Crtl) or *ARG2* targeting sgRNA-transduced K562 cells were subjected to puromycin selection to generate single cell clones. The clones were incubated at 21% or 1% O_2_ for 48 hours, and ARG2 expression was detected by western blot. (F) Arginase activity of the knockout clones was quantified as described for Fig 1D (average of 3 experiments). (G) The control and knockout clones were treated with nor−NOHA (1mM) at 21% or 1.5% O_2_ for 72 hours, and cell viability was determined by Annexin V/ 7−AAD staining (average of 3 experiments).

Although nor−NOHA is generally considered a specific arginase inhibitor and has been used in various studies [49, 50], its specificity has not been rigorously evaluated. To determine the biological significance of ARG2 in leukemic cells and validate the functional target of nor−NOHA, we knocked down ARG2 in various leukemic cell lines using both siRNA (transient) and shRNA (stable). Transient knockdown of ARG2 by siRNA in K562 had no effect on apoptotic cell death in the presence or absence of imatinib (Fig 5A). Similarly, stable knockdown of ARG2 in K562 and HL60 cells (Fig 5B; Part C and D in S2 Fig) also did not compromise cell viability (Fig 5C). Since RNA interference may not produce complete loss of gene function, and residual ARG2 protein levels may contribute to cell survival, we used the CRISPR/Cas9 system to genetically delete ARG2 and completely inactivate its arginase activity (Fig 5D). By using two different sgRNAs targeting the first exon of ARG2, we generated two clones (ARG2−KO#1 and #2, the genomic sequences are listed in the S5 Table) in which ARG2 protein expression and arginase activity were totally eliminated (Fig 5E and 5F). The ARG2−KO clones grew at similar rates as the control cells (data not shown) and, importantly, both ARG2−KO clones remained sensitive to nor−NOHA treatment (Fig 5G). Together, the above results suggest that the cytocidal effects of nor−NOHA are independent of ARG2 inhibition and are off-target.

### ARG2 knockout and nor−NOHA have distinct effects on cellular respiration

**Fig 6:**
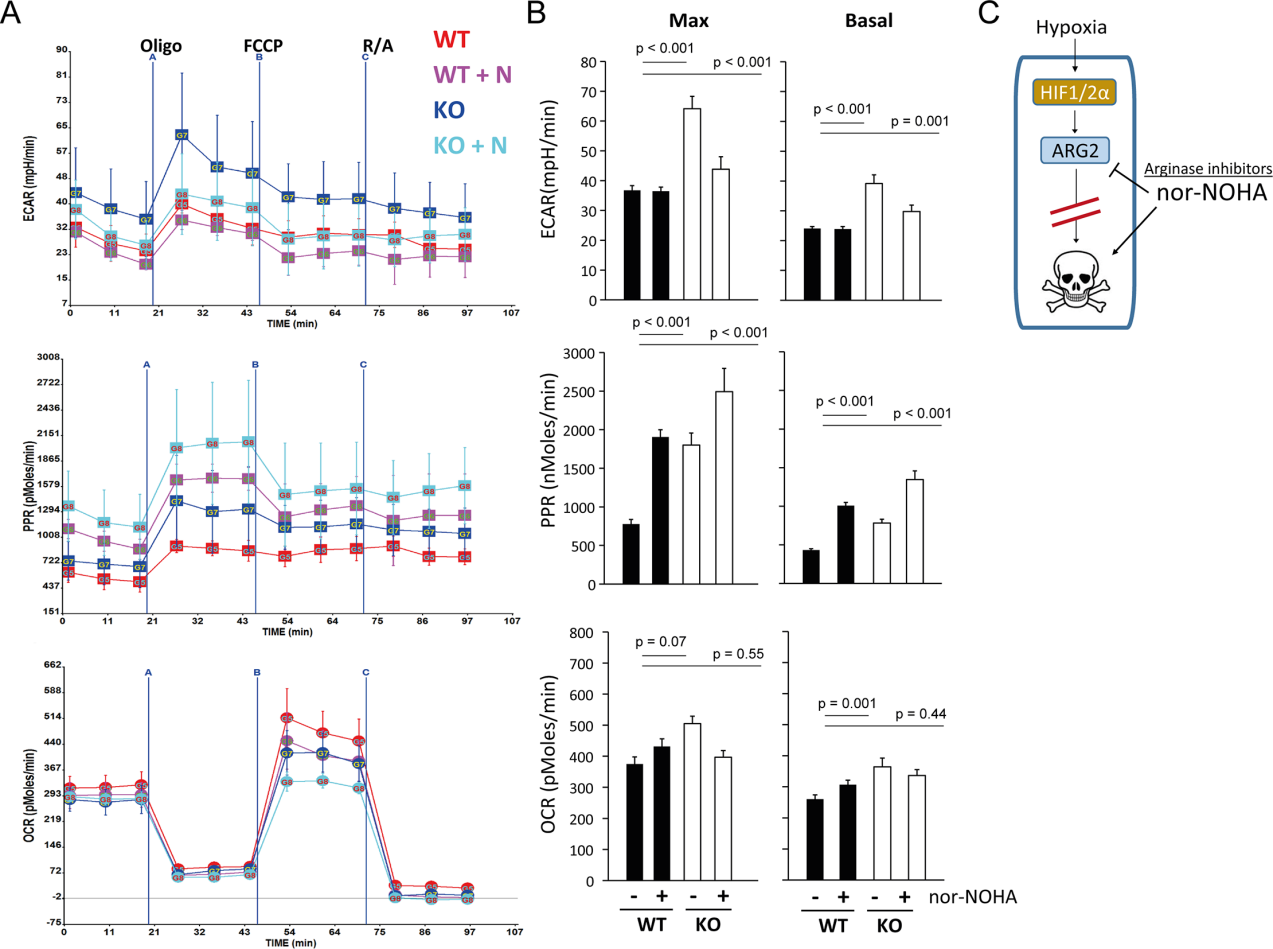
Genetic ablation of *ARG2* and nor−NOHA treatment produce distinct effects on cellular respiration. (A) Measurement of oxygen consumption rate (OCR), extracellular acidification rate (ECAR), and photon production rate (PPR) by the Seahorse XF−24 Analyser. The control (WT) or ARG2−KO K562 cells (two clones each) were treated with 1mM nor−NOHA for 24 hours before harvesting for analysis. Sequential injection of oligomycin (Oligo), carbonyl cyanide−4−(trifluoromethoxy) phenylhydrazone (FCCP), and rotenone & antimycin A (R/A) are as indicated. In each experiment, measurements were performed in quintuplicate for each cell line and condition. A representative graph of three independent experiments is shown. (B) Quantification of the maximal and basal OCR, ECAR and PPR levels. (C) Model depicting the relationship between HIFs, ARG2, and the cellular effects of nor−NOHA.

The above findings indicated that the cellular consequences of ARG2 ablation and nor-NOHA exposure were non-equivalent, and understanding their differential cellular effects might suggest potential mechanisms of nor-NOHA activity. Recent studies have shown that changes in cellular respiration are observed when arginase is overexpressed or its activity pharmacologically inhibited, including by nor−NOHA, and suggest that the arginases contribute to cellular respiration through their catalytic activity [26, 51]. Since our findings indicated that nor−NOHA’s effects were independent of arginase inhibition, we determined if the non-specific activity of nor−NOHA might be accounted for by its effects on cellular respiration. We therefore compared the effects of nor−NOHA and ARG2−KO on cellular respiration in K562 cells. We treated the ARG2−KO clones with nor−NOHA for 48 hours under normoxia and performed metabolic profiling where during this time the cells were largely viable (Part A in S4 Fig). Indicators of mitochondrial respiration, the oxygen-consumption rate (OCR), as well as measures of aerobic glycolysis, extracellular acidification rate (ECAR) and photon production rate (PPR), were measured using the Seahorse Analyzer. As compared to the control cells, ARG2-KO increased both the basal and maximal level of ECAR and PPR (Fig 6A and 6B). In contrast, nor−NOHA treatment increased the level of PPR but had no effect on ECAR in wild-type cells. In ARG2-KO clones, nor−NOHA was able to further alter PPR and ECAR despite the absence of its putative target ARG2, confirming differential effects of nor−NOHA and ARG2−KO on cellular respiration. We then went on to test the effect of re-expressing ARG2 in the ARG2−KO cells. Overexpression of the wildtype (WT) or arginase-dead mutant (H160F) of ARG2 increased OCR, ECAR and PPR in ARG2−KO cells (S4 Fig), suggesting that a non-enzymatic function of ARG2 might contribute to cellular respiration. There was no significant change of the OCR levels across all conditions. Taken together, the above results indicate that the effects of nor−NOHA on cellular respiration are at least in part, independent of ARG2 inhibition.

## Discussion

In the current study we addressed whether ARG2 represents a therapeutic target capable of distinguishing between normal and leukemic progenitors under hypoxic conditions. We found that hypoxia drives the expression of HIF1−α and HIF2−α, which in turn regulates ARG2 expression in a subset of leukemic cells. ARG2−expressing leukemic cells, including CML progenitors, were sensitive to the arginase inhibitor nor−NOHA under hypoxia. As such, nor−NOHA could overcome hypoxia-mediated imatinib resistance in CML progenitors. Unexpectedly, through extensive validation employing RNAi and CRISPR/Cas9 mutagenesis, we found that ARG2 loss-of-function was not responsible for the phenotypes produced by nor−NOHA treatment, although nor−NOHA did inhibit the arginase activity of ARG2 (Fig 6C). Our results highlight the possibility of using pharmacologic inhibitors to target pathways activated under defined physiologic conditions that favour the persistence and emergence of drug-resistant leukemic progenitors. However, our findings also emphasize the importance of vigorously validating potential therapeutic targets prior to pre-clinical or clinical drug development, which is a significant concern in the field [52–55].

Previously we identified ARG2 as a hypoxia regulated gene in CML progenitors [15]. Because of its high and specific expression in leukemic but not normal progenitors, frequent involvement in cancers and the availability of small molecule inhibitors, ARG2 was deemed to be a promising therapeutic target. The specific expression of ARG2 in a HIF1/2-α-regulated manner in leukemic cells, but not their normal counterparts, is consistent with the lineage restriction of arginase paralog expression in other cell types, e.g. HIF1−α induces ARG1 in macrophages [40] while HIF2−α induces ARG2 in endothelial [41] and epidermal cells [42]. In addition, the differential regulation of ARGs by HIFs in these other cell types suggests transcriptional regulation by other cofactors or epigenetic mechanisms beyond HIFs. The careful regulation of arginase expression suggests functional significance to the HIFs−ARG2 axis, but their precise role in leukemia remains unclear. ARG2 expression does not seem to modulate synthesis of nitric oxide, as none of the NOS isoforms were expressed (Part B in S1 Fig). The production of ornithine, a downstream product of arginase, does not seem to rely very much on ARG2, and ornithine alone does not have a major impact on cell survival (Fig 3C and 3D). The secretion of toxic ammonia, which is mainly eliminated by the urea cycle in cells, was not altered by nor−NOHA or ARG2−KO (data not shown). Taken together, although ARG2 is inhibited by nor−NOHA in the ARG2−expressing cells, ARG2 is not the *bona fide* target responsible for the observed phenotypes. However, our findings also indicate that the actual target(s) of nor−NOHA are likely very important for survival under hypoxia.

Arginases are best known for their role in mediating immunosuppression, and are thought to fulfil this function in the local cellular microenvironment as secreted enzymes. For example, in solid tumors, infiltrating myeloid derived suppressor cells (MSDSs) secrete ARG1 which acts to deplete extracellular arginine. Since arginine is essential for the proper functioning of T cells, the local depletion of arginine results in the suppression of T cell anti−tumor activity [46, 47, 56]. Apart from their role in infiltrating myeloid cells, recent reports suggest that tumour cells can also deplete arginine by expressing ARG2, either intra− or extracellularly [24, 57]. In this regard, arginase inhibitors have been used to restore arginine levels and thereby T cell-mediated immunosurveillance [57, 58]. In addition, endogenous arginases also contribute to cancer cell proliferation through the production of ornithine, an effect that can be overcome with arginase inhibitors [28, 29]. Because of these distinct roles, the specific effect of arginase inhibitors in different cellular contexts should be carefully elucidated, especially in the instance of *in vivo* studies, where both the extracellular (secreted) and intracellular (endogenous) effects of arginase may be operative. In the current work, we show that endogenous ARG2 is not required for survival of leukemic cells, and in contrast with previous studies [24], we could not detect significant levels of secreted ARG2 for the leukemic cells used in this study (data not shown). In summary, and through multiple genetic approaches, we conclude that ARG2 is dispensable for the survival of leukemic cells.

Because arginase has recently been linked to cellular respiration [26, 51], we also investigated whether changes in cellular respiration might contribute to the off-target effects of nor−NOHA. Although we did not measure cellular respiration under hypoxic conditions, we were still able to observe distinct effects of ARG−KO and nor−NOHA on respiration (Fig 6). We found that genetic ablation of ARG2 in ARG−KO cells produced different effects from nor−NOHA treatment. One possible explanation for these observations is that ARG2 protein might harbor non-enzymatic functions, and is consistent with a prior report describing the ability of ARG2 to impair autophagy in endothelial cells, independent of its arginase activity [59]. Importantly, we observed that overexpression of arginase-dead ARG2 mutants had similar effects on respiration as wild-type ARG2 (S4 Fig), further supporting the existence of a non-enzymatic function of arginase. In this regard, we observed upregulation of ARG2 upon nor−NOHA treatment (data not shown). This accumulation of ARG2 protein, despite being enzymatically inhibited, might be responsible for the observed effects on cellular respiration.

In summary, our results demonstrate the ability of small molecules to preferentially inhibit hypoxia-induced survival pathways in leukemia versus their normal counterparts, and underline the importance of stringent validation of putative therapeutic targets. This is especially important for drugs which may have pleiotropic effects such as nor−NOHA, and which have been extensively used in multiple disease and organ settings, including in cardiovascular diseases, asthma, and diabetes [45, 49, 50, 58, 60].

## Supporting information

S1 Fig Metabolism of arginine and the expression of arginine utilizing enzyme. (A) Schematic showing cellular metabolism of arginine. ARG; Arginase. NOS; nitric oxide synthase. ODC; ornithinse decarboxylase. OAT; ornithine aminotransferase. ARG competes with NOS for the common substrate arginine. (B) Expression of ARG and NOS family members in CML CP cells. CML CP CD34+ progenitors (n = 3) were treated with DMSO (control) or imatinib under 0.5% or 21% O_2_ for 24 or 96 hours. Expression profiling was done by microarray ([15], Illumina HumanHT-12 v4 beadchips; accession number GSE48294) and the absolute levels of expression were shown here (in log2, with background expression level of 4.3). (C) Expression levels of *ARG2* and *ARG1* in various cancer cell lines were obtained from the Cancer Cell Line Encyclopedia (CCLE).
(TIF)

S2 Fig HIFs regulate ARG2 expression. (A) ARG2 gene locus on UCSC genome browser. Of the two H3K27ac enriched regions near the ARG2 promoter, only one region contains putative HIF binding sites (highlighted in red) predicted by PROMO. (B) Knockdown of HIFs and ARGs by siRNA. K562 cells were transfected with control, HIF1-α, HIF2-α, ARNT (HIF1-β), ARG1 or ARG2 siRNA and were incubated under normoxia or hypoxia for 48 hours (n = 3). The corresponding transcript levels were measured by RT-qPCR. (C, D) Knockdown of ARG2 in HL60 cells reduces arginase activity in vitro and in vivo. HL60 cells were transduced with shRNA expressing vectors targeting Luc (control), HIF1-α or HIF2-α and the transduced cells were treated with 150 μM CoCl2 for 48 hours. Cells were harvested for in vitro arginase activity assays in (C) and the amount of urea in the cultured medium was quantified in (D) (average of 4 experiments).
(TIF)

S3 Fig Responses of individual CML samples towards nor-NOHA, Imatinib and hypoxia. Bar charts show colony numbers following treatment of 6 independent lots of primary patient CD34+ CML cells with combinations of normoxia (21% O2), hypoxia (1.5% O2), 0.5μM imatinib (IM) and/or 1mM nor−NOHA (NOHA) for 96 hours in colony forming assays. Numbers denote quantification of colonies for each condition.
(TIF)

S4 Fig ARG2 regulates cellular respiration independent of its arginase activity. (A) Viability of cells used for Seahorse metabolomics analysis. The cells were treated as described in Fig 6A, and were used for both Seahorse analysis (Fig 6A) and for cell viability assays by Annexin V/ 7-AAD staining (average of 3 experiments). (B) Overexpression of ARG2 and ARG2 mutant in CRISPR/Cas9 mediated ARG2 knockout K562 cells. Vectors expressing C-terminal GFP linked ARG2 (WT) or arginase-dead ARG2 (H160F) were transfected into ARG2 KO (#1) K562 cells. Transfected cells were cultured for 48 hours and harvested for western blotting (B), in vitro arginase assays (C) or metabolomics analysis using the Seahorse Analyser (D). For western blots, the expression of both GFP-tagged ARG2 (top bands) and untagged ARG2 (bottom bands) were detected.
(TIF)

S1 Table Antibodies used for western blotting. (XLSX)

S2 Table Primers used for RT-PCR. (XLSX)

S3 Table Sequence of the shRNA hairpins. (XLSX)

S4 Table Primers used for constructing lentiCRISPRv2 vectors. (XLSX)

S5 Table Genomic sequence of the ARG2-KO clones. (XLSX)
