# Supplementary figures and images for: The arginase inhibitor Nω−hydroxy−nor−arginine (nor−NOHA) induces apoptosis in leukemic cells specifically under hypoxic conditions but CRISPR/Cas9 excludes arginase 2 (ARG2) as the functional target
Source: PLoS One. 2018 Oct 11;13(10):e0205254. doi: 10.1371/journal.pone.0205254 (PMC6181325; doi:10.1371/journal.pone.0205254)

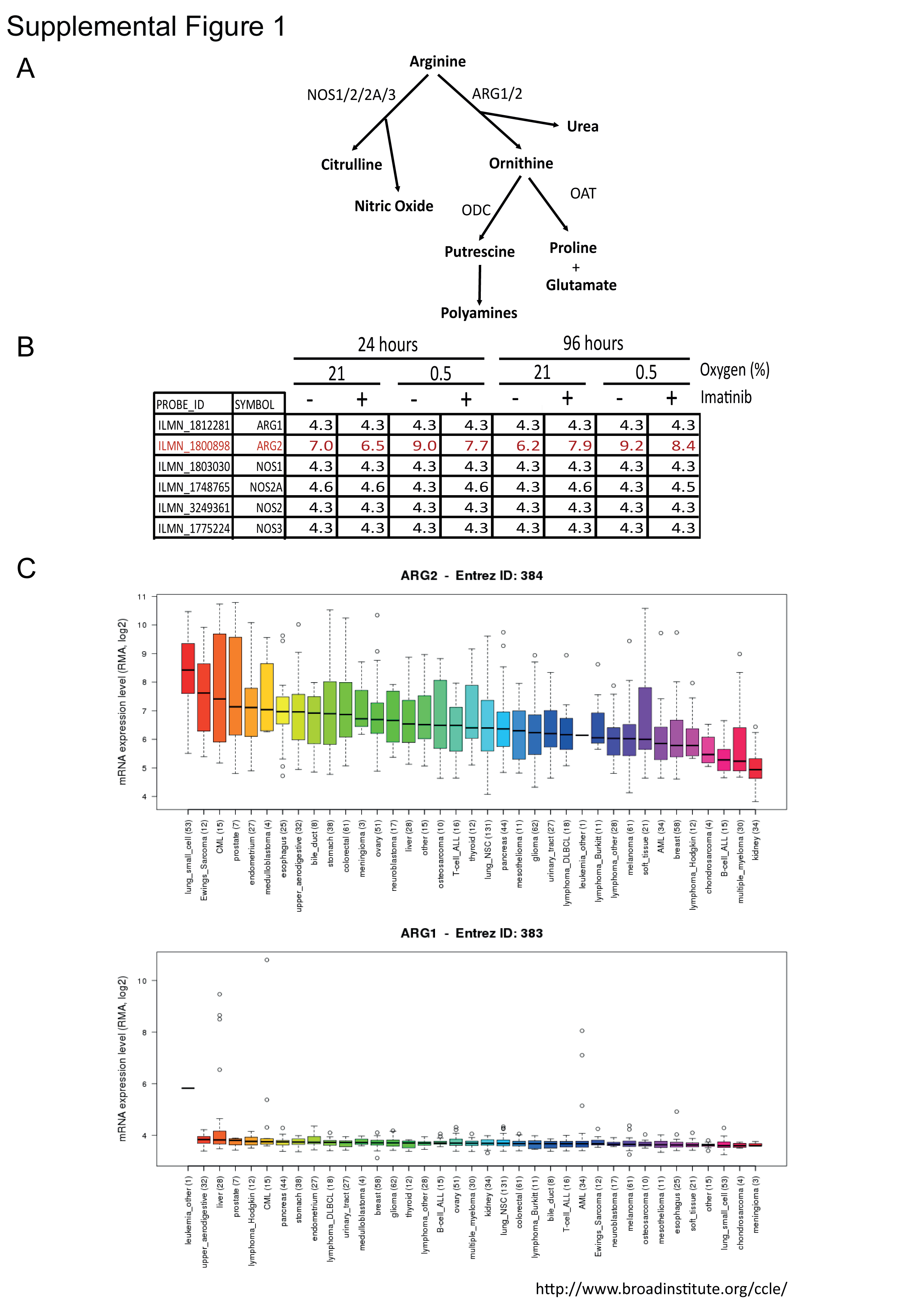

Supplement: S1 Fig — (A) Schematic showing cellular metabolism of arginine. ARG; Arginase. NOS; nitric oxide synthase. ODC; ornithinse decarboxylase. OAT; ornithine aminotransferase. ARG competes with NOS for the common substrate arginine. (B) Expression of ARG and NOS family members in CML CP cells. CML CP CD34+ progenitors (n = 3) were treated with DMSO (control) or imatinib under 0.5% or 21% O2 for 24 or 96 hours. Expression profiling was done by microarray ([15], Illumina HumanHT-12 v4 beadchips; accession number GSE48294) and the absolute levels of expression were shown here (in log2, with background expression level of 4.3). (C) Expression levels of ARG2 and ARG1 in various cancer cell lines were obtained from the Cancer Cell Line Encyclopedia (CCLE). (TIF) [file pone.0205254.s001.tif]

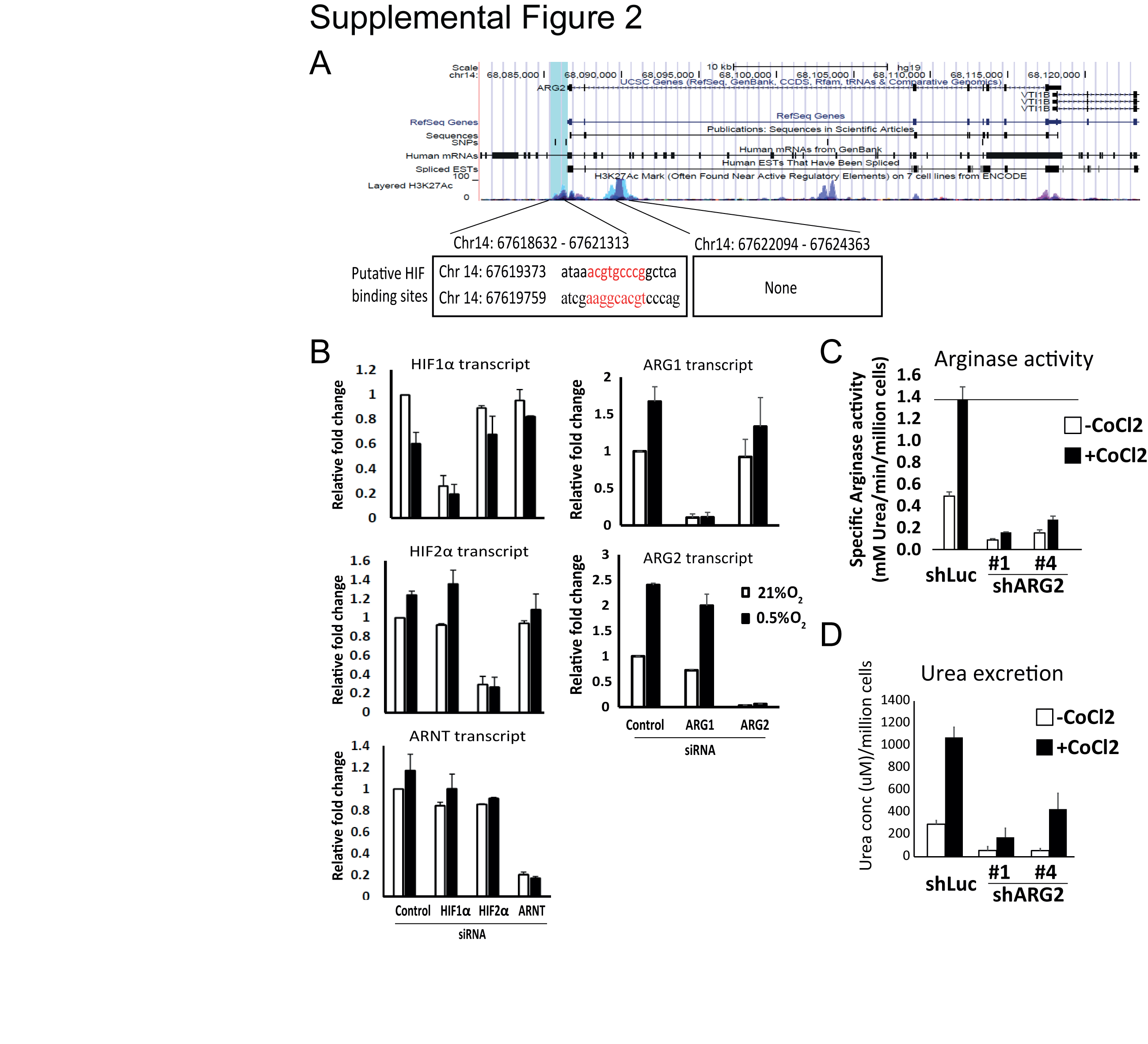

Supplement: S2 Fig — (A) ARG2 gene locus on UCSC genome browser. Of the two H3K27ac enriched regions near the ARG2 promoter, only one region contains putative HIF binding sites (highlighted in red) predicted by PROMO. (B) Knockdown of HIFs and ARGs by siRNA. K562 cells were transfected with control, HIF1-α, HIF2-α, ARNT (HIF1-β), ARG1 or ARG2 siRNA and were incubated under normoxia or hypoxia for 48 hours (n = 3). The corresponding transcript levels were measured by RT-qPCR. (C, D) Knockdown of ARG2 in HL60 cells reduces arginase activity in vitro and in vivo. HL60 cells were transduced with shRNA expressing vectors targeting Luc (control), HIF1-α or HIF2-α and the transduced cells were treated with 150 μM CoCl2 for 48 hours. Cells were harvested for in vitro arginase activity assays in (C) and the amount of urea in the cultured medium was quantified in (D) (average of 4 experiments). (TIF) [file pone.0205254.s002.tif]

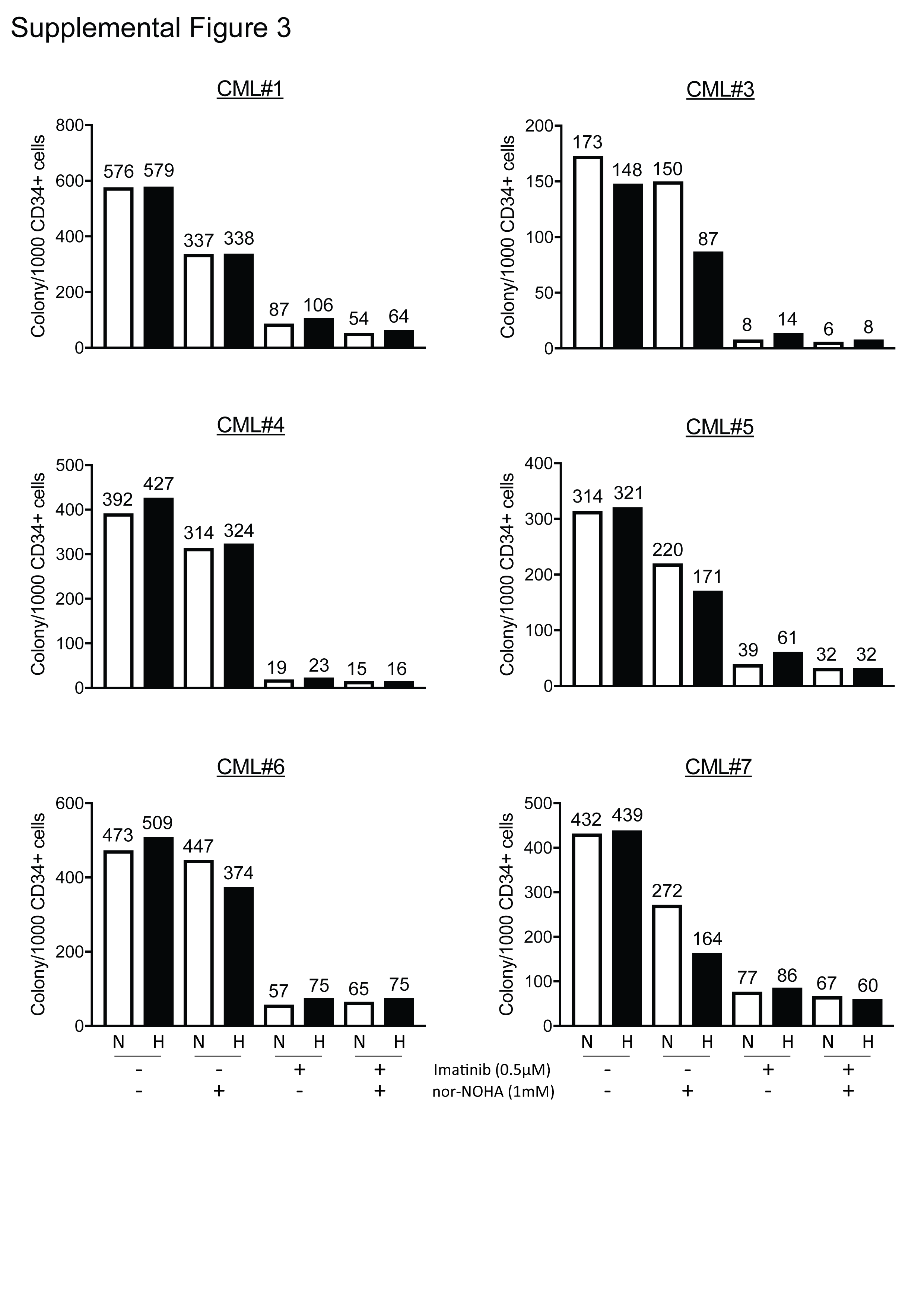

Supplement: S3 Fig — Bar charts show colony numbers following treatment of 6 independent lots of primary patient CD34+ CML cells with combinations of normoxia (21% O2), hypoxia (1.5% O2), 0.5μM imatinib (IM) and/or 1mM nor−NOHA (NOHA) for 96 hours in colony forming assays. Numbers denote quantification of colonies for each condition. (TIF) [file pone.0205254.s003.tif]

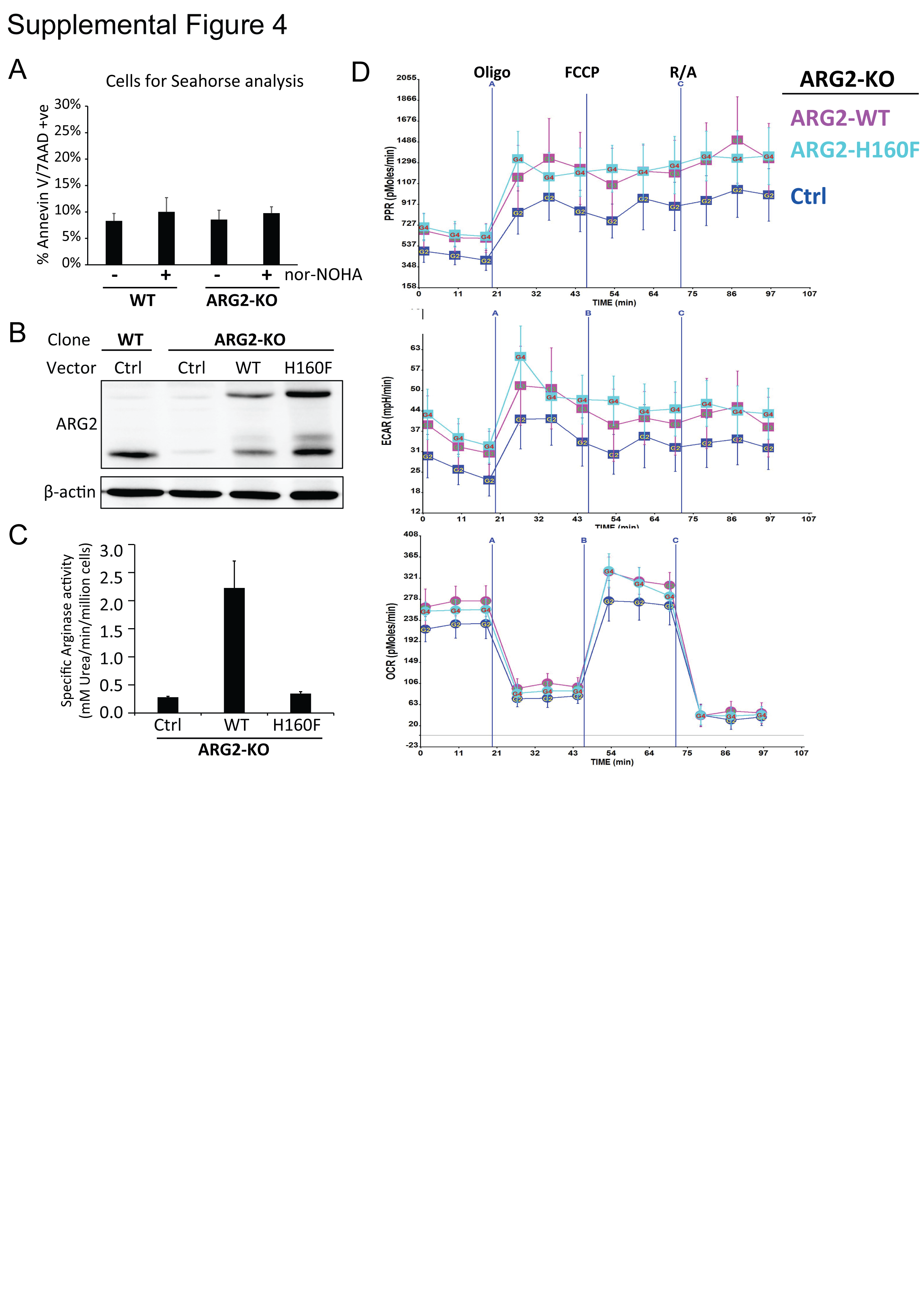

Supplement: S4 Fig — (A) Viability of cells used for Seahorse metabolomics analysis. The cells were treated as described in Fig 6A, and were used for both Seahorse analysis (Fig 6A) and for cell viability assays by Annexin V/ 7-AAD staining (average of 3 experiments). (B) Overexpression of ARG2 and ARG2 mutant in CRISPR/Cas9 mediated ARG2 knockout K562 cells. Vectors expressing C-terminal GFP linked ARG2 (WT) or arginase-dead ARG2 (H160F) were transfected into ARG2 KO (#1) K562 cells. Transfected cells were cultured for 48 hours and harvested for western blotting (B), in vitro arginase assays (C) or metabolomics analysis using the Seahorse Analyser (D). For western blots, the expression of both GFP-tagged ARG2 (top bands) and untagged ARG2 (bottom bands) were detected. (TIF) [file pone.0205254.s004.tif]
